# Matrix Production and Organization by Endothelial Colony Forming Cells in Mechanically Strained Engineered Tissue Constructs

**DOI:** 10.1371/journal.pone.0073161

**Published:** 2013-09-02

**Authors:** Nicky de Jonge, Dimitri E. P. Muylaert, Emanuela S. Fioretta, Frank P. T. Baaijens, Joost O. Fledderus, Marianne C. Verhaar, Carlijn V. C. Bouten

**Affiliations:** 1 Department of Biomedical Engineering, Eindhoven University of Technology, Eindhoven, The Netherlands; 2 Department of Nephrology and Hypertension, University Medical Center Utrecht, Utrecht, The Netherlands; 3 Institute for Complex Molecular Systems, Eindhoven University of Technology, Eindhoven, The Netherlands; University of California, Berkeley, United States of America

## Abstract

**Aims:**

Tissue engineering is an innovative method to restore cardiovascular tissue function by implanting either an *in vitro* cultured tissue or a degradable, mechanically functional scaffold that gradually transforms into a living neo-tissue by recruiting tissue forming cells at the site of implantation. Circulating endothelial colony forming cells (ECFCs) are capable of differentiating into endothelial cells as well as a mesenchymal ECM-producing phenotype, undergoing Endothelial-to-Mesenchymal-transition (EndoMT). We investigated the potential of ECFCs to produce and organize ECM under the influence of static and cyclic mechanical strain, as well as stimulation with transforming growth factor β1 (TGFβ1).

**Methods and Results:**

A fibrin-based 3D tissue model was used to simulate neo-tissue formation. Extracellular matrix organization was monitored using confocal laser-scanning microscopy. ECFCs produced collagen and also elastin, but did not form an organized matrix, except when cultured with TGFβ1 under static strain. Here, collagen was aligned more parallel to the strain direction, similar to Human Vena Saphena Cell-seeded controls. Priming ECFC with TGFβ1 before exposing them to strain led to more homogenous matrix production.

**Conclusions:**

Biochemical and mechanical cues can induce extracellular matrix formation by ECFCs in tissue models that mimic early tissue formation. Our findings suggest that priming with bioactives may be required to optimize neo-tissue development with ECFCs and has important consequences for the timing of stimuli applied to scaffold designs for both *in vitro* and *in situ* cardiovascular tissue engineering. The results obtained with ECFCs differ from those obtained with other cell sources, such as vena saphena-derived myofibroblasts, underlining the need for experimental models like ours to test novel cell sources for cardiovascular tissue engineering.

## Introduction

Cardiovascular diseases are a growing concern worldwide [1]. Surgical replacement of vascular structures such as heart valves and blood vessels is a commonly used therapy. While current cardiovascular replacements are effective [2], [3], they often rely on autologous tissues in the case of vascular replacement therapy or have significant shortcomings, including lack of remodeling and growth potential and the need for lifelong anticoagulation therapy. These concerns apply to pediatric patients in particular, necessitating reoperations throughout life as they outgrow their prostheses [4]. Tissue engineering (TE) has been proposed as a potential alternative to overcome these limitations. A tissue engineered cardiovascular construct can be produced *in vitro* by seeding human-derived autologous cells [5], [6] onto a biodegradable polymeric scaffold [7], followed by the application of biochemical and mechanical stimuli in bioreactors [8]. The final product is a living tissue [9], able to integrate, grow, and remodel with the patient upon scaffold degradation [7]. By using a pre-shaped scaffold, a tissue engineered construct can be obtained by seeding and conditioning human vena saphena cells (HVSCs) [8], [10]. HVSCs are often used for this approach because of their capacity to produce a strong extracellular matrix (ECM) network with distinct collagen fiber orientation [11], which is particularly critical when producing a mechanically functional substitute for the frequently affected load-bearing cardiovascular tissues. An important aim of functional TE is to gain control over the collagen orientation in a tissue, to create tissue substitutes that can remain mechanically functional and endure ongoing straining while in circulation.

The production of *in vitro* tissue engineered constructs, while promising, is time consuming and has limited scalability. *In situ* tissue engineering has been proposed as an innovative approach to obtain off-the-shelf available cardiovascular substitutes. Cardiovascular tissue function could be restored by the implantation of a mechanically functional but degradable scaffold that gradually transforms into a living tissue at the site of implantation by recruiting and stimulating circulating cells to form neo-tissue [12], [13]. For both *in vitro* and *in situ* cardiovascular tissue engineering approaches, major challenges are the selection of an appropriate cell source and to provide the right stimuli to guide the formation of an organized ECM to achieve and maintain tissue integrity and mechanical functioning.

Adult peripheral blood contains a rare population of circulating cells with endothelial colony forming capacity [14], high proliferative potential, and *in vivo* vasculogenic potential: Endothelial Colony Forming Cells (ECFCs). ECFCs have been proposed as a potential cell source for *in situ* cardiovascular TE [15], [16], [17] because they express haematopoietic markers as well as endothelial markers [18]. Laminar shear stress on ECFCs in 2D has been thoroughly investigated: it differentiates ECFCs towards the endothelial phenotype, as assessed by the anti-thrombogenic potential of ECFCs [19], [20] and the achievement of mature endothelial cell markers [21] with an arterial-like phenotype [22]. Moreover, ECFCs can change their differentiation pathway towards a mesenchymal phenotype, undergoing Endothelial-to-Mesenchymal-transition (EndoMT); this same process occurs during embryonic valvulogenesis and is of great interest for matrix production for TE, the focus of this paper.

A major stimulus that is present in cardiovascular tissues is cyclic strain. With *in situ* TE, for example, right after implantation of the scaffold, cells recruited to the scaffold *in vivo* will be under immediate and continuous cyclic strain, while producing the ECM that eventually should take over mechanical functionality from the scaffold. In adults, cyclic strain has been shown to induce EndoMT in valvular endothelial cells [23], [24], [25]. This process is characterized by a down-regulation of Tyrosine kinase with immunoglobulin-like domains (TIE2) [26] and an up-regulation of alpha smooth muscle actin (αSMA) [27]. An easily accessible circulating cell source capable of differentiating into endothelial cells as well as an ECM-producing cell type, such as ECFCs, would be of great potential for cardiovascular TE.

ECFCs can be isolated from adult peripheral blood, though their presence in umbilical cord blood is 15-fold higher [28], and they are a useful tool for *in vitro* experiments. In addition, in the context of *in vitro* TE, the high proliferative potential, ability to constitute an endothelial monolayer, and easily accessible source make these cells an interesting alternative to HVSCs, which have to be harvested invasively. Sheep ECFCs have been shown to respond to TGFβ1 with αSMA upregulation and tissue formation in pre-seeded *in vitro* cultured heart valves [15], [29], [30]. There is evidence of colony forming cells expressing both hematopoetic and endothelial markers homing to cell-free constructs in vivo in response to chemokines such as stromal cell derived factor 1α (SDF1α) [31]. However, little is known about the response of human ECFCs to mechanical strain with regard to their ability to create an oriented collagen matrix for load-bearing cardiovascular regeneration and TE.

Here we investigated the influence of static and cyclic mechanical strain on the production of an organized ECM by human ECFCs, by using a previously developed fibrin-based 3D tissue model to simulate neo-tissue formation [32]. As a control, HVSCs were used, since the response of HVSCs to mechanical strain has previously been well characterized [33], [34], [35], [36]. While native (cardiovascular) cells would be a good reference for an end-point comparison, we focused on the matrix organization in response to growth factors and mechanical strain.

Firstly, ECFC matrix production was assessed in the presence or absence of TGFβ1 and endothelial growth factors (eGFs), and in response to static or cyclic strain. We found that ECFCs were able to produce collagen and elastin. To test the hypothesis that transdifferentiated ECFCs can respond differently to strain (i.e.: by producing an oriented collagen matrix), cells were primed with TGFβ1, in medium without eGFs, prior to the application of static or cyclic strain. The ECM composition (collagen I, III, IV and elastin) was determined by mRNA expression analysis, flow cytometry, colorimetric elastin assay and histology, whereas ECM architecture was monitored using confocal laser scanning microscopy (CLSM) combined with live imaging. Furthermore, cell phenotype, or a change thereof, was assessed from endothelial (Von Willebrand factor (VWF) and TIE2) and mesenchymal (αSMA) markers.

## Materials and Methods

### I. Cell Culture and Experimental Design

#### Cell isolation and culture

ECFCs were isolated from human umbilical cord blood as previously described [37]. Approval was granted by a local ethics review board for the use of umbilical cord blood for stem cell research (01/230K, Medisch Ethisch Toetsings Commissie (METC), University Medical Center Utrecht). Written informed consent was given prior to collection of material. This consent procedure was approved by the METC. The study conforms with the principles outlined in the Declaration of Helsinki [38]. Three donors were used for the 2D studies; one donor was used for the 3D studies in triplo. Briefly, the mononuclear cell (MNC) fraction was isolated from whole blood using Ficoll-paque density gradient centrifugation (400 g. for 30 minutes). MNCs were plated on rat-tail collagen type I (BD Biosciences, Bedford, MA) coated six-well culture plates (Costar; Corning Incorporated, Corning, NY) in a final concentration of 2×10^7^ cells per well in endothelial growth medium (Medium A), consisting of endothelial basal medium (EBM-2) (Lonza) supplemented with 10% fetal bovine serum (FBS; Greiner Bio-One, Monroe, NC), 1% GlutaMax (Gibco, Carlsbad, CA), 1% penicillin streptomyocin (PenStrep; Lonza, Belgium) and Single Quots (EGM-2 BulletKit (CC-3162) containing hEGF, Hydrocortisone, GA-1000 (Gentamicin, Amphotericin-B), VEGF, hFGF-B, R^3^-IGF-1, Ascorbic Acid, Heparin), hereafter referred to as full medium. Medium was refreshed daily for the first 4 days. On day 7 the cells were trypsinized and plated on fresh collagen type I coated wells until colonies appeared. ECFC colonies were isolated and passaged at 90% confluency. ECFCs were used at passage 7–9.

**Figure 1:**
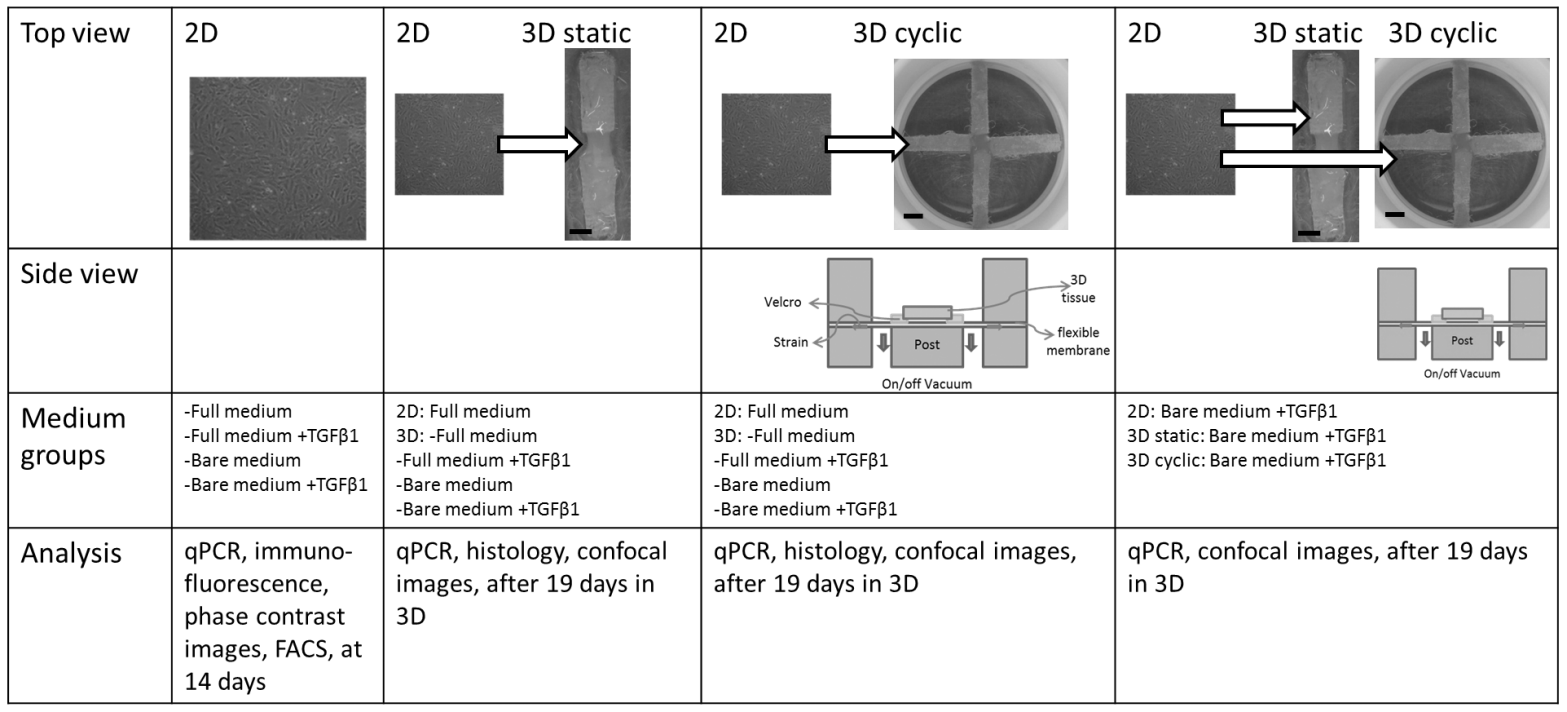
Schematic overview of the experimental set up. Top view shows 2D cell layer and 3D constructs on Bioflex plates with 2 (static strain protocol) or 4 (cyclic strain protocol) rectangular Velcro strips glued to the flexible membrane. Scale bar indicates 3 mm. Velcro strips leave space for cell-populated fibrin gels. Side views show schematic cross-section of the Flexcell setup used for cyclic strain application. When vacuum is applied, the flexible membrane is deformed over a rectangular loading post resulting in a uniaxial strain of the membrane and, thus, of the tissue. ECFCs were cultured in full medium, full medium +TGFβ1, bare medium and bare medium +TGFβ1. The bottom row shows analyses performed on the samples.

ECFCs were expanded in full endothelial growth medium (Figure 1). These cells were then cultured in 2D in different medium groups, referred to as; 1) full medium (as described before), 2) full medium +TGFβ1, 3) bare medium, and 4) bare medium +TGFβ1 (Figure 1). 5 ng/ml TGFβ1 is used for medium +TGFβ1. Bare medium consist of EBM-2, supplemented with 2% FBS for 2D culture and 5% FBS for 3D culture, 1% GlutaMax, 1% PenStrep and from Single Quots only Hydrocortisone, GA-1000, Ascorbic Acid and Heparin. 2% FBS was used in bare medium in 2D to slow down proliferation and allow transdifferentiation. 5% FBS was used in bare medium in 3D fibrin gels to avoid cell starvation. The effects of eGF depletion and TGFβ1 addition during exposure to static or cyclic strain were analyzed. To study the effect of sequential exposure to TGFβ1 and strain, primed ECFCs, defined as those cells obtained by 15 days of 2D culture of ECFCs in bare medium +TGFβ1, were subsequently cultured in 3D fibrin gels subjected to static or cyclic strain, cultured in bare medium +TGFβ1 as well.

Vascular-derived cells, for 2D and 3D culture of control groups, were acquired from one donor in accordance to Dutch guidelines for secondary use material. HVSCs were isolated from a human vena saphena magna following established protocols [39]. Culture medium consisted of advanced Dulbecco’s modified Eagle’s medium (a-DMEM; Gibco, Carlsbad, CA), supplemented with 10% FBS, 1% GlutaMax and 1% PenStrep and was refreshed every three days. The cells were passaged at 100% confluency. HVSCs were used at passage 7.

### II. Tissue Culture and Strain Application

#### Tissue culture

To investigate strain-induced matrix formation and organization by the cells, a previously developed system was used to host and strain 3D tissue constructs [32]. In brief, Velcro strips (8×3 mm) were glued to the flexible membranes of untreated 6-well Bioflex culture plates (Flexcell, McKeesport, PA) by using Silastic MDX4-4210 (Dow Corning, Midland, MI). For static culture, two Velcro strips per membrane were placed in opposite position to leave a rectangular space (4×5 mm) for a cell-populated fibrin gel of 80 µl (Figure 1). For cyclic culture, four rectangular Velcro strips were glued per membrane to form a cross shape and leaving a square space (5×5 mm) for a cell-populated fibrin gel of 120 µl (Figure 1). This procedure was chosen because uniaxial static culture induces collagen orientation [32], [40], while cyclic straining of biaxially constrained tissues induces strain avoidance with concomitant collagen orientation in myofibroblast-seeded tissues [40], [41]. Hence this set-up allows to study the mechano-response of cells and collagen organization in 3D constructs. The Bioflex plates were placed on a Flexcell FX-40001 system (Flexcell Int.) located in an incubator for prolonged strain application.

The fibrin gels were used as temporary matrix to create 3D cell-populated constructs with sufficient tissue integrity for strain application [32]. In brief, bovine fibrinogen and bovine thrombin were combined to produce final construct concentrations of 10 mg/ml fibrinogen, 10 IU/ml thrombin and 15×10^6^ cells/ml, based on methods used for TE heart valves [11]. ECFCs, primed ECFCs, and HVSCs (for control tissues) were mixed with the fibrin gel forming solution and plated immediately between the Velcro strips. The gels did not adhere to the flexible membrane (Figure 1). The constructs were incubated for 30 min at 37^o^C in a humidified 95/5% air/CO_2_ incubator to allow gelation before culture medium was added. For the first seven days of culture 1 mg/ml ε-Amino Caproic Acid (εACA; Sigma-Aldrich, St Louis, MO) was added to prevent fibrin degradation. Medium was replaced three times a week, and constructs were cultured up to 19 days in medium groups A-D. All medium groups for ECFCs contained EBM-2 with 1% GlutaMax and 1% Penstrep.

#### Straining protocols

The Flexcell system applies a strain to the Bioflex plates by applying a vacuum to the membrane, pulling the membrane over a loading post (Figure 1). Rectangular posts were used underneath the membranes, resulting in uniaxial strain applied to the tissues. Constructs in all experimental groups were first cultured statically for 5 days to achieve initial tissue integrity. Static strain constructs were then cultured for another 14 days while the tissue compacted between the Velcro constraints. Cyclic constructs were subjected to cyclic strain for the next 14 days, using a previously established intermittent uniaxial straining protocol [32]. This protocol consisted of an intermittent strain of a sine wave between 0% and 5%, at a frequency of 1 Hz for periods of 3 hours, alternated with 3 hours resting periods. Previous studies with HVSC-seeded constructs indicated that this protocol rapidly (<3 days) results in aligned collagen formation in 3D tissues [32], [42].

### III. Tissue Analysis

#### qPCR

Total RNA was isolated from the tissues using Trizol isolation according to the manufacturer’s protocol (Trizol, cat#10296-010, Invitrogen, Life Technologies Europe BV, Bleiswijk, the Netherlands). Samples were dissolved in Trizol and homogenized in a Precellys 24 tissue homogenizer (Precellys, Bertin Technologies, Aix-en Provence, France). cDNA was synthesized using iScript according to the manufacturer’s protocol (iScript, Cat#170-8891, Bio-Rad, Hercules, California, United States). qPCR was performed using the primers listed in Table 1 and data was analyzed using the delta-delta CT method, normalized to GAPDH expression.

**Table 1:** EndoMT markers and ECM genes and corresponding primer sequences used for qPCR.

Gene	Type	Function	Forward primer	Backward primer
VWF	Endothelial	Clotting factor	CCGATGCAGCCTTTTCGGA	TCTGGAAGTCCCCAATAATCGAG
TIE2	Endothelial	Angiopoietin receptor	TCCGCTGGAAGTTACTCAAGA	GAACTCGCCCTTCACAGAAATAA
αSMA	Mesenchymal	Cell contractility, structure	CAGGGCTGTTTTCCCATCCAT	GCCATGTTCTATCGGGTACTTC
Elastin	Extracellular matrix	Elastic part	CGCCCAGTTTGGGTTAGTTC	CACCTTGGCAGCGGATTTTG
Collagen type I	Extracellular matrix	Load bearing part	GTCGAGGGCCAAGACGAAG	CAGATCACGTCATCGCACAAC
Collagen type III	Extracellular matrix	Fibrillar collagen	TTGAAGGAGGATGTTCCCATCT	ACAGACACATATTTGGCATGGTT
Collagen type IV	Extracellular matrix	Basal lamina	AGATAAGGGTCCAACTGGTGT	ACCTTTAACGGCACCTAAAATGA

#### Immunohistochemistry and histology

Immunohistochemistry was performed on cells cultured in monolayers on coverglasses. Cells were fixed with 10% formalin (Sigma-Aldrich) for 15 minutes and permeabilized with 0.1% Triton-X-100 (Merck, Germany) in PBS (Sigma-Aldrich) for 10 minutes. To block non-specific binding, cells were incubated for 1 h in 2% w/v solution of bovine serum albumin (BSA, Roche) in PBS, followed by 2 hours incubation with the primary antibody solution in 0.5% BSA. After washing, the cells were incubated with the secondary antibody solutions and, subsequently, with DAPI. Samples were mounted onto rectangular microscope slides using Mowiol mounting medium and analyzed by fluorescent microscopy (Axiovert 200, Carl Zeiss). All used antibodies are listed in Table 2. Histology was performed on 3D tissue constructs. The constructs were first washed in phosphate-buffered saline (PBS), embedded in Tissue-Tek (Sakura, the Netherlands) and then frozen in isopentane at −80°C. Transverse cross-sections of 10 µm were cut and stained with Verhoeff van Gieson (VvG) for detection of collagen and elastic fibers and visualized by light microscopy.

**Table 2:** Antibodies and corresponding secondary antibodies used for immunofluorescence.

Marker	Primary antibody	Secondary antibody
CD31	Mouse anti-human IgG1 (Dako)	Alexa fluor 555 goat anti-mouse IgG1 (Invitrogen)
	1∶200 v/v	1∶300 v/v
Collagen type 1	Rabbit anti-human IgG (Abcam)	Alexa fluor 555 goat anti-rabbit IgG(H+L) (Invitrogen)
	1∶300 v/v	1∶300 v/v
Collagen type III	Rabbit anti-human IgG (Abcam)	Alexa fluor 555 donkey anti-rabbit IgG(H+L) (Invitrogen)
	1∶200 v/v	1∶300 v/v
Collagen type IV	Mouse anti-human IgG1 (Abcam)	
	1∶200 v/v	1∶300 v/v
αSMA	Mouse anti-human IgG2a (Sigma)	Alexa Fluor 488 goat anti-mouse IgG2a (Invitrogen)
	1∶500 v/v	1∶300 v/v

#### Live confocal microscopy

To visualize the organization of cells and collagen fibers in the engineered constructs, confocal microscopy was performed as described previously [43]. In short, samples were labeled by Cell Tracker Orange (CTO; Invitrogen Molecular Probes) and CNA35-OG488 (CNA) [44], to fluorescently stain cell cytoplasm and collagen respectively. CTO and CNA are excitable at 466 nm and 520 nm. Scans were made using an inverted Zeiss Axiovert 200 microscope (Carl Zeiss, Oberkochen, Germany) coupled to an LSM 510 Meta (Carl Zeiss) laser scanning microscope.

#### Flow Cytometry

Cells cultured in 2D with medium A and D were trypsinized and allowed to recover for 10 minutes at room temperature in EBM-2 medium with 2% serum. After washing with PBS the cells were fixated, permeabilized and stained with LeukoPerm (cat# YSRTBUF09, Gentaur Belgium BV, Kampenhout, Belgium) according to the manufacturers protocol. Briefly: cells were washed in PBS and stained for the extracellular marker CD31, washed and fixated for 15 minutes in fixation buffer. Following washing steps in PBS the cells were permeabilized and stained for the intracellular marker αSMA for 30 minutes. After final washing steps the cells were analyzed on a FACS CANTO II flow cytometer and data was analyzed using FACSDIVA software.

#### Elastin quantification

The Fastin Elastin Assay (Biocolor, UK) was used to quantify elastin content of tissue samples, after qPCR was performed. To extract proteins from tissue samples in Trizol, protein isolation was performed using 2-propanol (Merck) and proteins were washed using guanidine hydrochlorine (0.3 M, Sigma), according to manufacturer’s protocol. To extract insoluble α-elastin from tissue samples, several extraction steps were used, incubating tissue samples with oxalic acid anhydrous (0.25 M, Fluka Chemie, St. Louis, MO) for 1 hour at 100°C. The extract is treated as described in the manufacturer’s protocol, and dye intensity is measured at 513 nm. For three tissue samples per experimental condition, elastin content was calculated in µg per tissue sample.

#### Statistical analysis

Data is shown as means +/− standard deviation. Statistical analysis was performed using GraphPad Prism 5.0 (GraphPad Software, San Diego, California, USA) and p values <0.05 were considered significant. T-test was used to analyze flow cytometry data and one-way ANOVA with post-hoc Tukey analyses were used to analyze qPCR and Elastin Fastin data.

## Results

**Figure 2:**
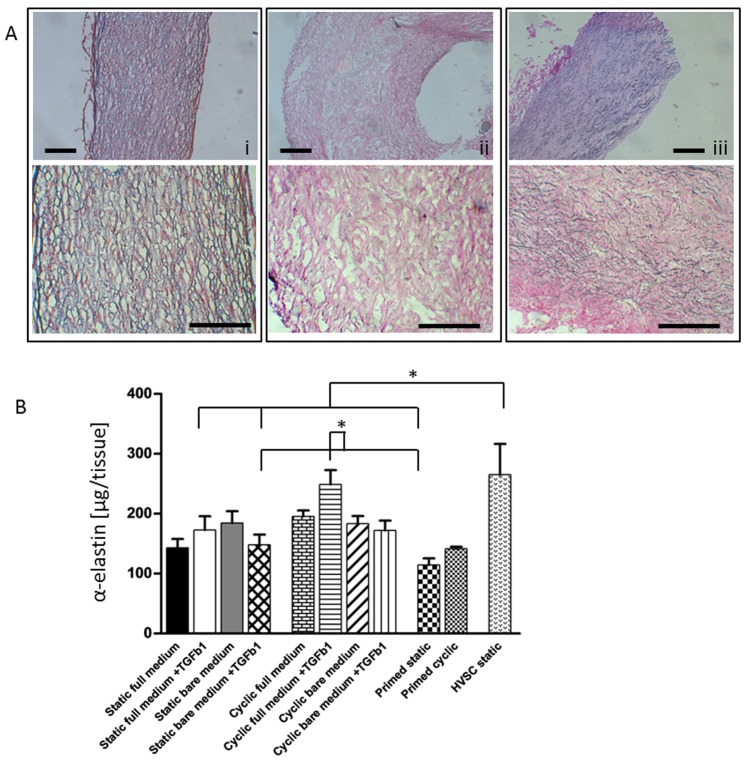
Elasin and collagen staining on slices of 3D constructs (A) and quantification of elastin (B). Representative images are shown for the Verhoeff van Gieson staining (A), with elastin in blue-black and collagen in red. On this global scale results for all ECFC groups (all medium groups, static or cyclic strain) were similar. (i) ECFCs showed both collagen and elastin, (ii) HVSCs showed only collagen. (iii) Native human tissue was used as a positive control for collagen and elastin. Scale bar 200 µm. Elastin quantification by Fastin Elastin assay (B) showed significant differences, where cyclic full medium+TGFβ1 is significantly higher than primed static and static bare medium+TGFβ1, and HVSC static is significantly higher than primed static, static bare medium +TGFβ1 and static full medium +TGFβ1; * indicates p<0.05, N = 3.

**Figure 3:**
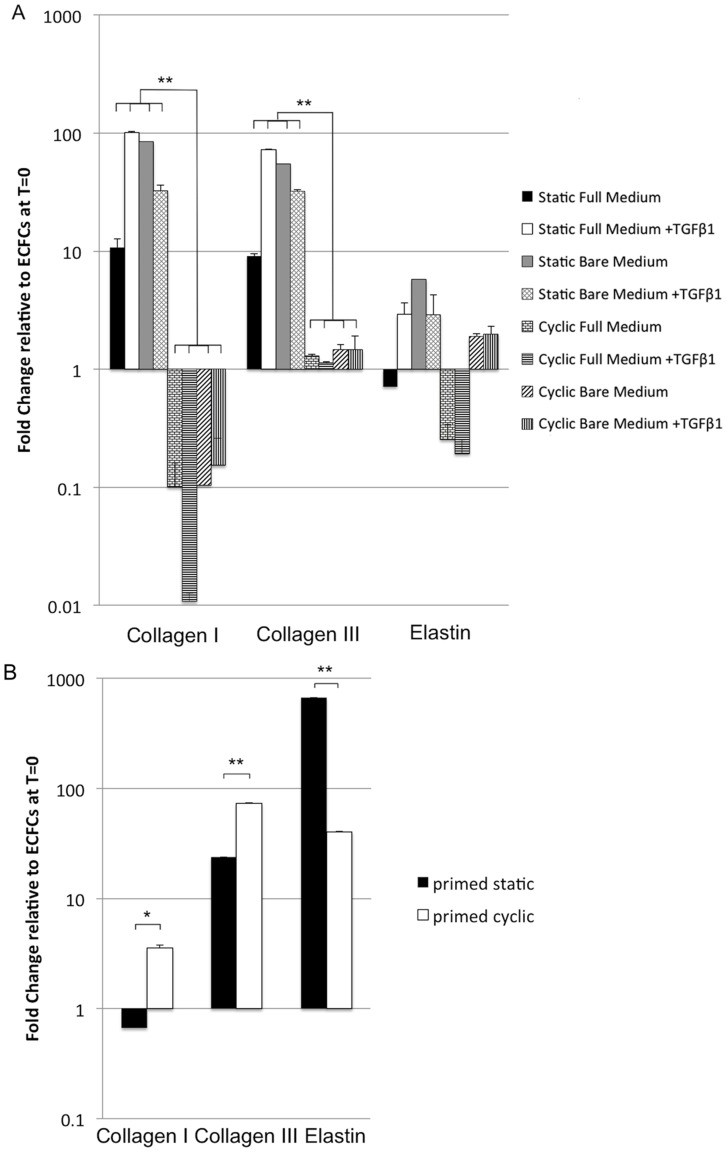
qPCR of ECFCs and primed ECFCs under static and cyclic strain in 3D. Expression of ECM genes is shown relative to primed ECFCs before seeding into the 3D gels. (A) Cyclic strain significantly decreased expression of collagen type I and III in ECFCs for all medium groups. The expression of collagen IV was not detected. (B) Cyclic strain significantly upregulated expression of collagen type I and III in ECFCs primed with TGFβ1. Elastin expression was reduced in these cells in response to cyclic strain. * = p<0.05, ** = p<0.01, N = 3.

**Figure 4:**
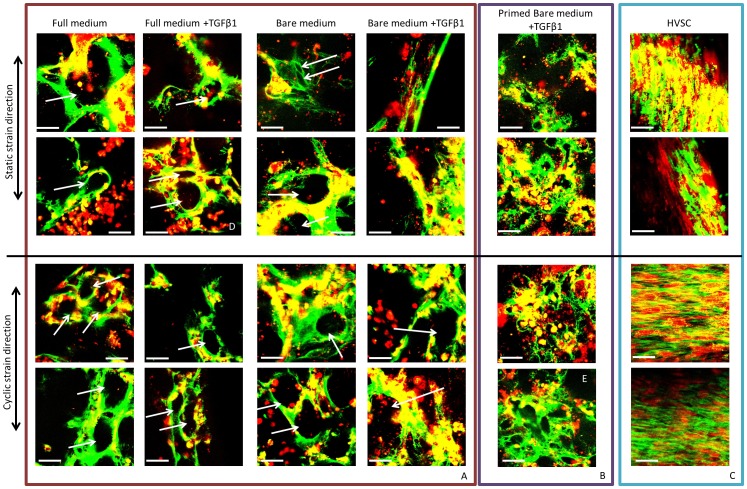
Confocal images of details (duplo) of ECFC- and HVSC-seeded constructs. Constructs were cultured for 19 days in all medium groups and under static or cyclic strain. Cells are shown in red, collagen in green. (A) ECFCs formed cell colonies and produced tubular collagen structures (indicated by white arrows) independent of medium type or strain, except when cultured in bare medium +TGFβ1 under static strain, when a slightly more alinged matrix was observed. (B) Primed ECFCs distributed more homogenously throughout the tissue and deposite collagen more homogenously throughout the tissue. (C) HVSCs showed distinct collagen alignment and strain responsiveness: collagen aligned parallel to the uniaxial strain direction in case of static straining and orthogonal to the strain direction in the cyclically loaded, biaxially constrained tissues. Scale bar indicates 50 µm. The difference in tubular structure for ECFCs and more homogeneous collagen distribution for primed ECFCs is shown in Movies for image (D) and (E).

Culturing ECFCs in 3D under static strain resulted in an upregulation of gene expression for collagen I and III relative to ECFCs in 2D. In addition, histology showed that ECFCs had produced collagen as well as elastin throughout the tissue constructs (Figure 2A). No clear elastin structures were found in HVSC tissue constructs, though the elastin quantification (Figure 2B) showed that α-elastin monomers was produced in amounts similar to ECFCs. The strained ECFCs showed no differences in elastin content for the different conditions (Figure 2B). The expression of collagen I and III in cyclically strained constructs was significantly reduced relative to the static controls (Figure 3A), indicating an inhibitory effect of cyclic strain. These results were consistent for all medium groups, indicating collagen I and III gene expression was mostly related to the type of strain applied, rather than to growth factors in the culture medium. The confocal images showed that, contrary to HVSCs, ECFCs deposited collagen in tubular structures, except when cultured in the presence of TGFβ1 and the absence of eGFs under static strain (Figure 4A). Here, collagen was aligned to strain direction, similar to HVSC-seeded controls (Figure 4C). Tubular structures extend through the construct, as shown in a 3D reconstruction of confocal images (Movie S1, corresponding to the 2D image in Figure 4D).

**Figure 5:**
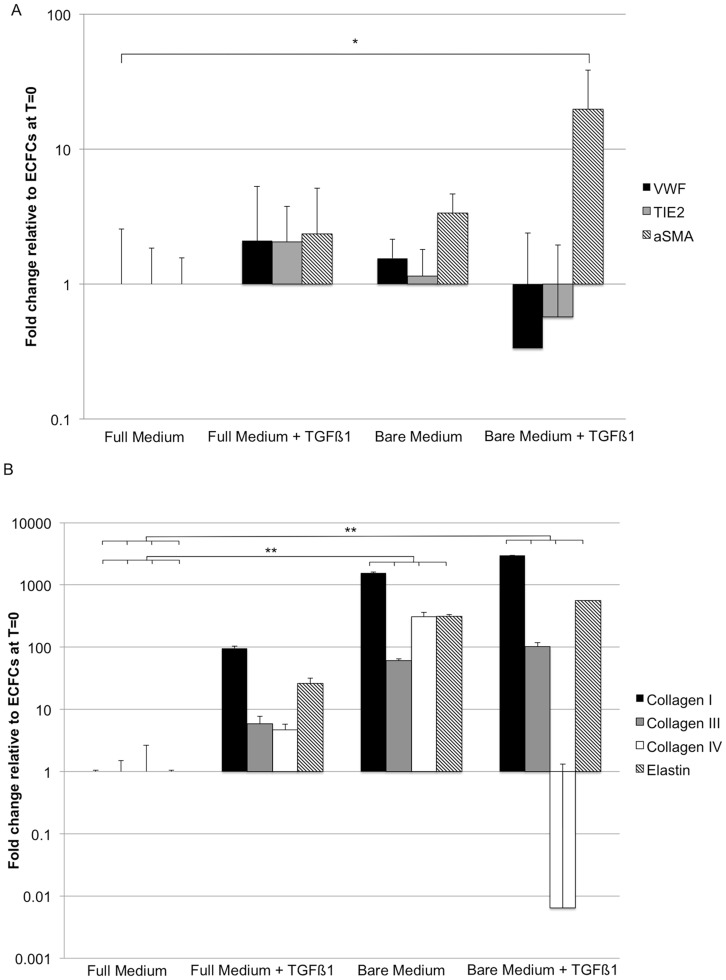
qPCR of ECFCs cultured in 2D for 14 days on collagen type I coating. (A) Full medium +TGFβ1 and bare medium showed an increased αSMA expression compared to ECFCs of full medium. Bare medium +TGFβ1 showed a light downregulation of endothelial markers VWF and TIE2, while the expression of αSMA was significantly upregulated. (B) With full medium +TGFβ1 and bare medium with or withour TGFβ1 a significant increase in collagen type I, III and elastin was detected. A decrease in collagen IV expession was found for collagen type IV. * = p<0.05, ** = p<0.01, N = 3.

**Figure 6:**
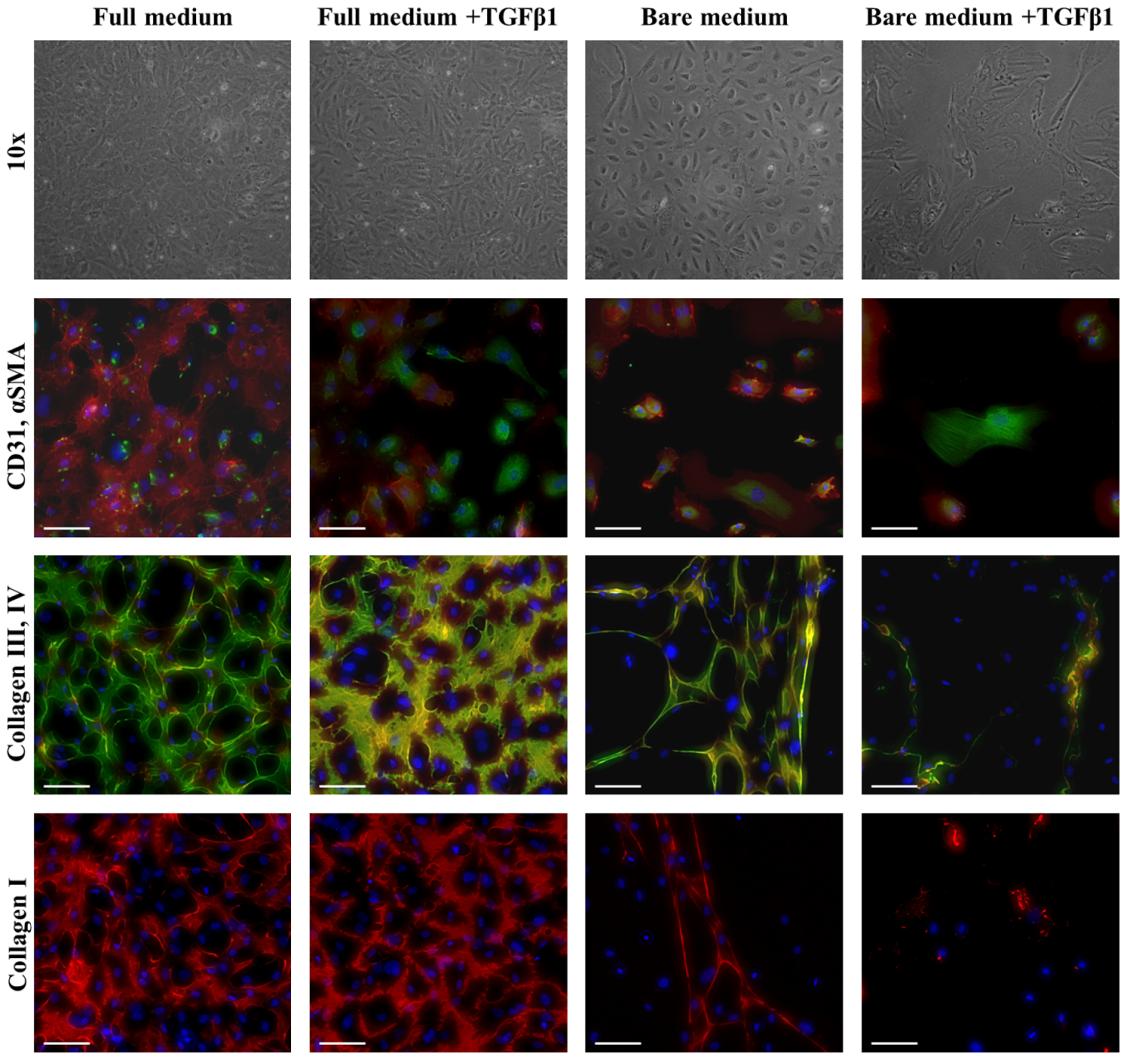
Phase contrast and immunofluorescent images of ECFCs on coverglasses in different media. Phase contrast pictures, after 14 days of culture, at 10×magnification show the complete change in ECFC morphology in bare medium +TGFβ1, where cells are elongated and they have lost the cobblestone morphology, still retained by ECFCs cultured in the other media. Immunostaining for CD31 (red), αSMA (green) and DAPI (blue), confirmed the differences observed by optical imaging, with loss of CD31 in part of the cells cultured in full medium +TGFβ1 and bare medium +TGFβ1, and αSMA stress fibers only detected in bare medium +TGFβ1. Immunostaining for collagen type III (green), IV (red) and DAPI (blue) showed a decrease in matrix production in bare medium with or without TGFβ1. This result was also confirmed for collagen type I (red). The scalebar represents 100 µm.

We tested whether priming ECFCs by TGFβ1 addition and eGF depletion could initiate transdifferentiation into an ECM-expressing cell type that is strain-responsive. To confirm endothelial phenotype, ECFCs were analyzed by flow cytometry (Figure S1): ECFCs were positive for endothelial markers CD105, CD31 and KDR, while being negative for leukocyte markers CD14 and CD45, indicating a mature endothelial phenotype [28]. After two weeks of 2D culture in bare medium +TGFβ1, the mRNA levels of endothelial markers (VWF and TIE-2) were downregulated, while the expression of αSMA was significantly upregulated compared to full medium (Figure 5A). The expression of the ECM genes collagen I, III and elastin was upregulated significantly in bare medium, with or without TGFβ1 (Figure 5B), while a downregulation in collagen type IV expression was found. Immunohistochemistry showed that, while αSMA stress fibers were present, CD31 also remained detectable (Figure 6). However, in the presence of TGFβ1 the localization of the CD31 protein shifted from the cell membrane to a more cytosolic distribution (Figure 6), indicating a loss of intercellular adhesion. Culturing ECFCs in a 3D environment in these culture media under static or cyclic strain resulted in no reduction of endothelial markers or increase in αSMA for any medium condition (Figure S2).

To assess the response to sequential exposure to TGFβ1, in bare medium, in 2D followed by mechanical strain in 3D, the cells were cultured for two weeks in bare medium+TGFβ1. 20% of the cells were positive for αSMA and 100% for CD31 as measured by flow cytometry, confirming qPCR (Figure S3) and immunohistochemistry results (Figure 6). The primed ECFCs were then all cultured under static or cyclic strain conditions in 3D, in bare medium +TGFβ1. These cells distributed more homogenously throughout the fibrin tissue (Figure 4B, Movie S2), similar to HVSCs (Figure 4C), and deposited collagen in a more homogenous pattern (Figure 4B). Collagen I and III expression was significantly increased and elastin was decreased in response to cyclic strain as compared to static strain (Figure 3B). No Collagen IV expression was observed. Although lowered by cyclic strain, the elastin gene expression in static constructs remained higher in primed ECFCs in 3D compared to primed ECFCs in 2D. There was, however, no alignment of the produced collagen fibers in response to strain directionality (Figure 4B). In contrast, HVSCs, frequently used in pre-seeded tissue TE, showed a higher gene expression for collagen I (Figure S4) and a clear alignment of matrix in response to strain direction (Figure 4C).

## Discussion

Cardiovascular tissue engineered constructs have to function under continuous hemodynamic loads. Here, we analyzed the response of a potential new cell source for cardiovascular tissue engineering, ECFCs, to combined and sequential stimulation with cyclic mechanical strain and growth factors. Although sequential stimulation of cells has been studied for the production of ECM for *in vitro* cardiovascular tissue engineering applications [5], [29], [42], [45], [46], the production and orientation of matrix by circulating progenitor cells in response to cyclic strains has received little attention. Our results showed that EFCFs in 3D constructs produced organized collagen and elastin, albeit in a different fashion than the control HVSCs. At the gene expression level cyclic straining of the ECFC constructs – even in the presence of TGFβ1– was shown to inhibit ECM expression as compared to static strain conditions after 19 days of culture. Interestingly, priming ECFCs with TGFβ1 prior to exposure to strain was shown to upregulate the expression of ECM genes in a mechanically active environment.

One of the main drawbacks of HVSCs as a cell source for vascular structures is their lack of elastin network formation. Histology showed that in spite of elastin protein production (quantified with the Fastin Elastin assay), HVSCs were not able to polymerize and form an elastin network at the tissue level, whereas ECFCs did produce elastin in a more organized manner, visualized by histology (Figure 2A). There was no significant influence on the amount of elastin protein produced by ECFCs regardless of culture medium or straining. This contrasts with the downregulation of elastin mRNA found in response to cyclic strain in all conditions. This may be explained by deposition of elastin proteins prior to downregulation of the elastin gene. A similar effect was seen by Seliktar et al., who studied elastin production by smooth muscle cells and myofibroblasts in collagen gels: On mRNA level, elastin upregulation by these cells was detected up to 8 days, but upregulation was less in strained gels compared to unstrained gels [47].

In 2D static ECFC culture, removal of eGFs and addition of TGFβ1 induced a change of phenotype towards a tissue producing phenotype on a gene expression level. Interestingly, in 3D fibrin gels under static strain conditions ECFCs showed an upregulation of ECM genes, regardless of TGFβ1 or eGF depletion, indicating that placement in the 3D fibrin environment by itself promotes ECM gene expression. However, this upregulation was not seen when the ECFCs in their 3D environment were cyclically strained, indicating that cyclic strain, such as found in the cardiovascular system, may not be beneficial for matrix production by ECFCs. This response contrasts with the behavior of HVSCs, which align themselves and their produced matrix in response to strain, underlining the relevance of mechanic 3D in-vitro model systems to test potential cell sources for TE.

In contrast to untreated ECFCs, TGFβ1-primed ECFCs gave a more homogeneous distribution of cells and collagen throughout the 3D constructs and an upregulation of ECM gene expression for collagen I and III in cyclic compared to static strain. These results underline the importance of the 3D *in vitro* environment and an appropriate sequence of exposure to biochemical and mechanical stimuli for matrix production, relevant for the engineering of load-bearing cardiovascular tissues. Compared to the tubular structures that developed with non-primed ECFC cultures (Movie S1), the ECM structure formed by TGFβ1-primed ECFCs may result in better load-bearing tissues. These cells produced a more homogeneous distributed matrix, and even though 2D images seem to show tubular structures with smaller diameters (Figure 4B), in the 3D reconstruction (Movie S2) it can be appreciated that these structures do not extend throughout the construct. This may provide improved mechanical properties to the tissue, though the collagen structure does not resemble the load-bearing, anisotropic collagen network comparable to the aligned collagen fibers formed by HVSCs (Figure 4C).

Our data suggest that optimizing the timing between attracting circulating cells to the scaffold and exposing them to biochemical cues may be of critical importance for cardiovascular TE. The timing could potentially be influenced in an *in situ* TE context by incorporating TGFβ1 directly into the scaffold material with tunable slow release systems [48], [49]. The idea of combining the effects of bioactive stimuli to induce EndoMT with scaffolds with load-bearing mechanical properties was explored by Sewell-Loftin et al. [50]. In addition, it was previously reported that after seeding sheep ECFCs into a 3D scaffold, expression of αSMA and ECM genes was increased [29], [30]; though no mechanical straining was applied in these studies. Consistent with our results, these cells were reported to express both αSMA and CD31 after TGFβ1 stimulation.

In our study only a limited number of cells primed with TGFβ1 responded with αSMA gene upregulation. A non-uniform transdifferentiation of ECFCs into tissue producing cells may be due to a variation in differentiation potential [51], and could also explain why the TGFβ1-primed ECFCs were not strain responsive and unable to align themselves and the deposited collagen fibers. A limitation to our experiments is that we only studied neo-tissue formation at one time point in its early phase. How the tissue develops and matures over longer periods of time should be carefully examined in *in vivo* studies.

A clear advantage of ECFCs compared to HVCS is their circulating nature, making them a more easily accessible cell source for both *in situ* and *in vitro* tissue engineering strategies. In addition ECFCs are capable of constituting a mature endothelial lining after culture [19], [20], [21], [22] and stem from a circulating progenitor precursor. Though ECFCs and primed ECFCs are the result of extensive *in vitro* treatments, they also represent a model system for circulating progenitor cells capable of populating single-step *in situ* vascular grafts and heart valves [16], [52], [53]. The lower circulating progenitor cell number in peripheral blood and specific attraction of ECFCs into such grafts remain challenging and important hurdles [54], [55]. Potential strategies could draw inspiration from studies showing increased mobilization in response to factors released following ischemic damage [56]. It has been shown that with use of VEGF the number of circulating endothelial progenitor cells (EPCs) can be increased [55]. It is likely that any cell-free graft will attract a mixed population of cells, and strategies are actively being explored to preferentially attract progenitor cells [54], [55]. Attraction of a selective cell population has been extensively reviewed by de Mel et al. [55], where they discuss a strategy where endothelium derived macromolecules are used to assist in specific EPC adhesion.

In conclusion, ECFCs are an interesting potential cell source for cardiovascular tissue engineering since they can both form an endothelial lining on the surface of an engineered tissue and have the potential to produce ECM. We showed that ECFCs can produce ECM in a 3D construct, but not in response to cyclic strain to create an oriented, load-bearing collagen organization, needed for cardiovascular functioning. This implies that cyclic mechanical strain, such as found in the cardiovascular system may inhibit ECM formation by ECFCs. TGFβ1-priming of ECFCs before exposure to strain, however, enhanced matrix production and matrix organization, suggesting that sequential exposure to these soluble and mechanical factors is paramount to achieve the desired cellular response and tissue formation. Additional knowledge on how to control and time these factors may assist in the development of scaffold materials that guide the development of functional cardiovascular tissue. It should be noted, however, that the observed responses may be highly cell-type specific. Our results indicate that the well-studied response to mechanical strain of HVSCs in terms of matrix production and remodeling cannot be extrapolated to ECFCs. 3D *in vitro* model systems, such as presented here, offer the possibility to systematically investigate cell responses and tissue formation in dependency of cell source and environmental stimuli.

## Supporting Information

Figure S1 **Characterization of ECFCs by (A) flow cytometry of cell type markers and microscopy (B).** ECFCs do not express the leukocyte markers CD14 or CD45, retain some CD34 expression but not CD133, and express the endothelial markers CD105 (Endoglin), CD31 (PECAM), CD144 (VE-Cadherin) and KDR (VEGFR-2). A monolayer of ECFCs shows a typical cobblestone pattern in vitro (magnification 4x).
(TIF)

Figure S2 **qPCR of ECFCs cultured in 3D in all medium groups under static or cyclic strain.** Expression of EndoMT genes is shown relative to ECFCs before seeding into the 3D gels. Medium groups and static or cyclic strain did not result in any significant altering of the gene expression of VWF, TIE2 and αSMA. N = 3
(TIF)

Figure S3 **Flow cytometry of ECFCs before and after priming with TGFβ1 and depletion of eGFS for 15 days.** (A) The percentage of cells positive for αSMA increased to 20%. The percentage of cells positive for CD31 remained 100%. (B) Representative scatterplots of ECFCs before priming with TGFβ1 and (C) after priming with TGFβ1.
(TIF)

Figure S4 **qPCR of HVSCs and pre-treated ECFCs cultured in 2D.** Expression of ECM genes is shown relative to ECFCs, cultured in 2D with full medium. Pre-treated ECFCs show an up-regulation of collagen I compared to ECFCs. HVSCs on the other hand, express collagen type I significantly more than pre-treated ECFCs do. No significant differences were found comparing HVSCs with pre-treated ECFCs for collagen III, IV and elastin. ** = p<0.01, N = 3.
(TIF)

Movie S1 **A 3D reconstruction of tubular structures formed by ECFCs in 3D fibrin gels.** Confocal scans are converted to a 3D reconstruction showing a typical example. The tubular structures extend through the construct. Cells are shown in red, collagen in green. Scale bar indicates 50 µm.
(AVI)

Movie S2 **A 3D reconstruction of more homogeneous collagen distribution by primed ECFCs in 3D fibrin gels.** Confocal scans are converted to a 3D reconstruction showing a typical example. No tubular structures can be observed. Cells are shown in red, collagen in green. Scale bar indicates 50 µm.
(AVI)

## References

[1] Rippel RA, Ghanbari H, Seifalian AM (2012) Tissue-engineered heart valve: future of cardiac surgery. World J Surg 36: 1581–1591. 22395345 10.1007/s00268-012-1535-y

[2] Holmes DR Jr, Mack MJ, Kaul S, Agnihotri A, Alexander KP, et al (2012) 2012 ACCF/AATS/SCAI/STS expert consensus document on transcatheter aortic valve replacement: Developed in collabration with the American Heart Association, American Society of Echocardiography, European Association for Cardio-Thoracic Surgery, Heart Failure Society of America, Mended Hearts, Society of Cardiovascular Anesthesiologists, Society of Cardiovascular Computed Tomography, and Society for Cardiovascular Magnetic Resonance. J Thorac Cardiovasc Surg 144: e29–84. 22898522 10.1016/j.jtcvs.2012.03.001

[3] Reardon MJ (2012) Cost-Effectiveness Analysis of TAVR. Methodist Debakey Cardiovasc J 8: 26–28. 10.14797/mdcj-8-2-26 PMC3405797 22891125

[4] Rosengart TK, Feldman T, Borger MA, Vassiliades TA Jr, Gillinov AM, et al (2008) Percutaneous and minimally invasive valve procedures: a scientific statement from the American Heart Association Council on Cardiovascular Surgery and Anesthesia, Council on Clinical Cardiology, Functional Genomics and Translational Biology Interdisciplinary Working Group, and Quality of Care and Outcomes Research Interdisciplinary Working Group. Circulation 117: 1750–1767. 18332270 10.1161/CIRCULATIONAHA.107.188525

[5] van Geemen D, Riem Vis PW, Soekhradj-Soechit S, Sluijter JP, de Liefde-van Beest M, et al (2011) Decreased mechanical properties of heart valve tissue constructs cultured in platelet lysate as compared to fetal bovine serum. Tissue Eng Part C Methods 17: 607–617. 21284560 10.1089/ten.TEC.2010.0556

[6] Shin’oka T, Matsumura G, Hibino N, Naito Y, Watanabe M, et al (2005) Midterm clinical result of tissue-engineered vascular autografts seeded with autologous bone marrow cells. J Thorac Cardiovasc Surg 129: 1330–1338. 15942574 10.1016/j.jtcvs.2004.12.047

[7] Mol A, Smits AI, Bouten CV, Baaijens FP (2009) Tissue engineering of heart valves: advances and current challenges. Expert Rev Med Devices 6: 259–275. 19419284 10.1586/erd.09.12

[8] Thomas LV, Lekshmi V, Nair PD (2012) Tissue engineered vascular grafts - Preclinical aspects. Int J Cardiol. 10.1016/j.ijcard.2012.09.069 23040078

[9] Hinz B, Gabbiani G (2003) Mechanisms of force generation and transmission by myofibroblasts. Curr Opin Biotechnol 14: 538–546. 14580586 10.1016/j.copbio.2003.08.006

[10] Mol A, Driessen NJ, Rutten MC, Hoerstrup SP, Bouten CV, et al (2005) Tissue engineering of human heart valve leaflets: a novel bioreactor for a strain-based conditioning approach. Ann Biomed Eng 33: 1778–1788. 16389526 10.1007/s10439-005-8025-4

[11] Mol A, van Lieshout MI, Dam-de Veen CG, Neuenschwander S, Hoerstrup SP, et al (2005) Fibrin as a cell carrier in cardiovascular tissue engineering applications. Biomaterials 26: 3113–3121. 15603806 10.1016/j.biomaterials.2004.08.007

[12] De Visscher G, Vranken I, Lebacq A, Van Kerrebroeck C, Ganame J, et al (2007) In vivo cellularization of a cross-linked matrix by intraperitoneal implantation: a new tool in heart valve tissue engineering. Eur Heart J 28: 1389–1396. 17244642 10.1093/eurheartj/ehl422

[13] Roh JD, Sawh-Martinez R, Brennan MP, Jay SM, Devine L, et al (2010) Tissue-engineered vascular grafts transform into mature blood vessels via an inflammation-mediated process of vascular remodeling. Proc Natl Acad Sci U S A 107: 4669–4674. 20207947 10.1073/pnas.0911465107 PMC2842056

[14] Vranken I, De Visscher G, Lebacq A, Verbeken E, Flameng W (2008) The recruitment of primitive Lin(−) Sca-1(+), CD34(+), c-kit(+) and CD271(+) cells during the early intraperitoneal foreign body reaction. Biomaterials 29: 797–808. 18022690 10.1016/j.biomaterials.2007.10.041

[15] Sales VL, Mettler BA, Engelmayr GC Jr, Aikawa E, Bischoff J, et al (2010) Endothelial progenitor cells as a sole source for ex vivo seeding of tissue-engineered heart valves. Tissue Eng Part A 16: 257–267. 19698056 10.1089/ten.tea.2009.0424 PMC2811057

[16] Mazzolai L, Bouzourene K, Hayoz D, Dignat-George F, Liu JW, et al (2011) Characterization of human late outgrowth endothelial progenitor-derived cells under various flow conditions. J Vasc Res 48: 443–451. 21625177 10.1159/000324844

[17] Fioretta ES, Fledderus JO, Burakowska-Meise EA, Baaijens FP, Verhaar MC, et al (2012) Polymer-based scaffold designs for in situ vascular tissue engineering: controlling recruitment and differentiation behavior of endothelial colony forming cells. Macromol Biosci 12: 577–590. 22566363 10.1002/mabi.201100315

[18] Mund JA, Estes ML, Yoder MC, Ingram DA Jr, Case J (2012) Flow cytometric identification and functional characterization of immature and mature circulating endothelial cells. Arterioscler Thromb Vasc Biol 32: 1045–1053. 22282356 10.1161/ATVBAHA.111.244210 PMC3306529

[19] Yang Z, Tao J, Wang JM, Tu C, Xu MG, et al (2006) Shear stress contributes to t-PA mRNA expression in human endothelial progenitor cells and nonthrombogenic potential of small diameter artificial vessels. Biochem Biophys Res Commun 342: 577–584. 16488398 10.1016/j.bbrc.2006.01.172

[20] Yang Z, Wang JM, Wang LC, Chen L, Tu C, et al (2007) In vitro shear stress modulates antithrombogenic potentials of human endothelial progenitor cells. J Thromb Thrombolysis 23: 121–127. 17221326 10.1007/s11239-006-9045-0

[21] Yamamoto K, Takahashi T, Asahara T, Ohura N, Sokabe T, et al (2003) Proliferation, differentiation, and tube formation by endothelial progenitor cells in response to shear stress. J Appl Physiol 95: 2081–2088. 12857765 10.1152/japplphysiol.00232.2003

[22] Obi S, Yamamoto K, Shimizu N, Kumagaya S, Masumura T, et al (2009) Fluid shear stress induces arterial differentiation of endothelial progenitor cells. J Appl Physiol 106: 203–211. 18988767 10.1152/japplphysiol.00197.2008

[23] Balachandran K, Alford PW, Wylie-Sears J, Goss JA, Grosberg A, et al (2011) Cyclic strain induces dual-mode endothelial-mesenchymal transformation of the cardiac valve. Proc Natl Acad Sci U S A 108: 19943–19948. 22123981 10.1073/pnas.1106954108 PMC3250145

[24] Ghazanfari S, Tafazzoli-Shadpour M, Shokrgozar MA (2009) Effects of cyclic stretch on proliferation of mesenchymal stem cells and their differentiation to smooth muscle cells. Biochem Biophys Res Commun 388: 601–605. 19695226 10.1016/j.bbrc.2009.08.072

[25] Michalopoulos E, Knight RL, Korossis S, Kearney JN, Fisher J, et al (2011) Development of methods for studying the differentiation of human mesenchymal stem cells under cyclic compressive strain. Tissue Eng Part C Methods 18: 252–262. 22047076 10.1089/ten.tec.2011.0347 PMC3311877

[26] Medici D, Kalluri R (2012) Endothelial-mesenchymal transition and its contribution to the emergence of stem cell phenotype. Semin Cancer Biol. 10.1016/j.semcancer.2012.04.004 PMC3422405 22554794

[27] Krenning G, Moonen JR, van Luyn MJ, Harmsen MC (2008) Vascular smooth muscle cells for use in vascular tissue engineering obtained by endothelial-to-mesenchymal transdifferentiation (EnMT) on collagen matrices. Biomaterials 29: 3703–3711. 18556062 10.1016/j.biomaterials.2008.05.034

[28] Ingram DA, Mead LE, Tanaka H, Meade V, Fenoglio A, et al (2004) Identification of a novel hierarchy of endothelial progenitor cells using human peripheral and umbilical cord blood. Blood 104: 2752–2760. 15226175 10.1182/blood-2004-04-1396

[29] Sales VL, Engelmayr GC Jr, Mettler BA, Johnson JA Jr, Sacks MS, et al (2006) Transforming growth factor-beta1 modulates extracellular matrix production, proliferation, and apoptosis of endothelial progenitor cells in tissue-engineering scaffolds. Circulation 114: I193–199. 16820571 10.1161/CIRCULATIONAHA.105.001628

[30] Dvorin EL, Wylie-Sears J, Kaushal S, Martin DP, Bischoff J (2003) Quantitative evaluation of endothelial progenitors and cardiac valve endothelial cells: proliferation and differentiation on poly-glycolic acid/poly-4-hydroxybutyrate scaffold in response to vascular endothelial growth factor and transforming growth factor beta1. Tissue Eng 9: 487–493. 12857416 10.1089/107632703322066660

[31] Segers VF, Tokunou T, Higgins LJ, MacGillivray C, Gannon J, et al (2007) Local delivery of protease-resistant stromal cell derived factor-1 for stem cell recruitment after myocardial infarction. Circulation 116: 1683–1692. 17875967 10.1161/CIRCULATIONAHA.107.718718

[32] de Jonge N, Kanters FM, Baaijens FP, Bouten CV (2012) Strain-induced Collagen Organization at the Micro-level in Fibrin-based Engineered Tissue Constructs. Ann Biomed Eng. 10.1007/s10439-012-0704-3 23184346

[33] Ingber DE (2009) From cellular mechanotransduction to biologically inspired engineering: 2009 Pritzker Award Lecture, BMES Annual Meeting October 10, 2009. Ann Biomed Eng 38: 1148–1161. 10.1007/s10439-010-9946-0 PMC2913424 20140519

[34] Hinz B (2007) Formation and function of the myofibroblast during tissue repair. J Invest Dermatol 127: 526–537. 17299435 10.1038/sj.jid.5700613

[35] Huang H, Kamm RD, Lee RT (2004) Cell mechanics and mechanotransduction: pathways, probes, and physiology. Am J Physiol Cell Physiol 287: C1–11. 15189819 10.1152/ajpcell.00559.2003

[36] Wang JH, Grood ES (2000) The strain magnitude and contact guidance determine orientation response of fibroblasts to cyclic substrate strains. Connect Tissue Res 41: 29–36. 10826706 10.3109/03008200009005639

[37] Broxmeyer HE, Srour E, Orschell C, Ingram DA, Cooper S, et al (2006) Cord blood stem and progenitor cells. Methods Enzymol 419: 439–473. 17141066 10.1016/S0076-6879(06)19018-7

[38] World Medical Association Declaration of Helsinki. Recommendations guiding physicians in biomedical research involving human subjects. Cardiovasc Res 35: 2–3. 9302340

[39] Schnell AM, Hoerstrup SP, Zund G, Kolb S, Sodian R, et al (2001) Optimal cell source for cardiovascular tissue engineering: venous vs. aortic human myofibroblasts. Thorac Cardiovasc Surg 49: 221–225. 11505318 10.1055/s-2001-16113

[40] Weidenhamer NK, Tranquillo RT (2013) Influence of Cyclic Mechanical Stretch and Tissue Constraints on Cellular and Collagen Alignment in Fibroblast-Derived Cell Sheets. Tissue Eng Part C Methods. 10.1089/ten.tec.2012.0423 PMC3603568 23126441

[41] Foolen J, Deshpande VS, Kanters FMW, Baaijens FPT (2012) The influence of matrix integrity on stress-fiber remodeling in 3D. Biomaterials 33: 7508–7518. 22818650 10.1016/j.biomaterials.2012.06.103

[42] Rubbens MP, Mol A, Boerboom RA, Bank RA, Baaijens FPT, et al (2009) Intermittent straining accelerates the development of tissue properties in engineered heart valve tissue. Tissue Engineering Part A 15: 999–1008. 18795866 10.1089/ten.tea.2007.0396

[43] Boerboom RA, Krahn KN, Megens RT, van Zandvoort MA, Merkx M, et al (2007) High resolution imaging of collagen organisation and synthesis using a versatile collagen specific probe. J Struct Biol 159: 392–399. 17572104 10.1016/j.jsb.2007.04.008

[44] Krahn KN, Bouten CV, van Tuijl S, van Zandvoort MA, Merkx M (2006) Fluorescently labeled collagen binding proteins allow specific visualization of collagen in tissues and live cell culture. Anal Biochem 350: 177–185. 16476406 10.1016/j.ab.2006.01.013

[45] Mann BK, Schmedlen RH, West JL (2001) Tethered-TGF-beta increases extracellular matrix production of vascular smooth muscle cells. Biomaterials 22: 439–444. 11214754 10.1016/s0142-9612(00)00196-4

[46] Syedain ZH, Tranquillo RT (2011) TGF-beta1 diminishes collagen production during long-term cyclic stretching of engineered connective tissue: implication of decreased ERK signaling. J Biomech 44: 848–855. 21251657 10.1016/j.jbiomech.2010.12.007 PMC3061833

[47] Seliktar D, Nerem RM, Galis ZS (2003) Mechanical strain-stimulated remodeling of tissue-engineered blood vessel constructs. Tissue Eng 9: 657–666. 13678444 10.1089/107632703768247359

[48] Pham QP, Sharma U, Mikos AG (2006) Electrospinning of polymeric nanofibers for tissue engineering applications: a review. Tissue Eng 12: 1197–1211. 16771634 10.1089/ten.2006.12.1197

[49] Sengupta D, Heilshorn SC (2010) Protein-engineered biomaterials: highly tunable tissue engineering scaffolds. Tissue Eng Part B Rev 16: 285–293. 20141386 10.1089/ten.teb.2009.0591

[50] Sewell-Loftin MK, Chun YW, Khademhosseini A, Merryman WD (2011) EMT-inducing biomaterials for heart valve engineering: taking cues from developmental biology. J Cardiovasc Transl Res 4: 658–671. 21751069 10.1007/s12265-011-9300-4 PMC3310168

[51] Paruchuri S, Yang JH, Aikawa E, Melero-Martin JM, Khan ZA, et al (2006) Human pulmonary valve progenitor cells exhibit endothelial/mesenchymal plasticity in response to vascular endothelial growth factor-A and transforming growth factor-beta2. Circ Res 99: 861–869. 16973908 10.1161/01.RES.0000245188.41002.2c PMC2810464

[52] Jordan JE, Williams JK, Lee SJ, Raghavan D, Atala A, et al (2012) Bioengineered self-seeding heart valves. J Thorac Cardiovasc Surg 143: 201–208. 22047685 10.1016/j.jtcvs.2011.10.005

[53] Andukuri A, Sohn YD, Anakwenze C, Lim DJ, Brott B, et al.. (2012) Enhanced human endothelial progenitor cell adhesion and differentiation by a bioinspired multifunctional nanomatrix. Tissue Eng Part C Methods. 10.1089/ten.tec.2012.0312 PMC3603564 23126402

[54] Timmermans F, Plum J, Yoder MC, Ingram DA, Vandekerckhove B, et al (2009) Endothelial progenitor cells: identity defined? J Cell Mol Med 13: 87–102. 19067770 10.1111/j.1582-4934.2008.00598.x PMC3823038

[55] de Mel A, Jell G, Stevens MM, Seifalian AM (2008) Biofunctionalization of biomaterials for accelerated in situ endothelialization: a review. Biomacromolecules 9: 2969–2979. 18831592 10.1021/bm800681k

[56] Massa M, Rosti V, Ferrario M, Campanelli R, Ramajoli I, et al (2005) Increased circulating hematopoietic and endothelial progenitor cells in the early phase of acute myocardial infarction. Blood 105: 199–206. 15345590 10.1182/blood-2004-05-1831

