# Supplementary figures and images for: Matrix Production and Organization by Endothelial Colony Forming Cells in Mechanically Strained Engineered Tissue Constructs
Source: PLoS One. 2013 Sep 2;8(9):e73161. doi: 10.1371/journal.pone.0073161 (PMC3759389; doi:10.1371/journal.pone.0073161)

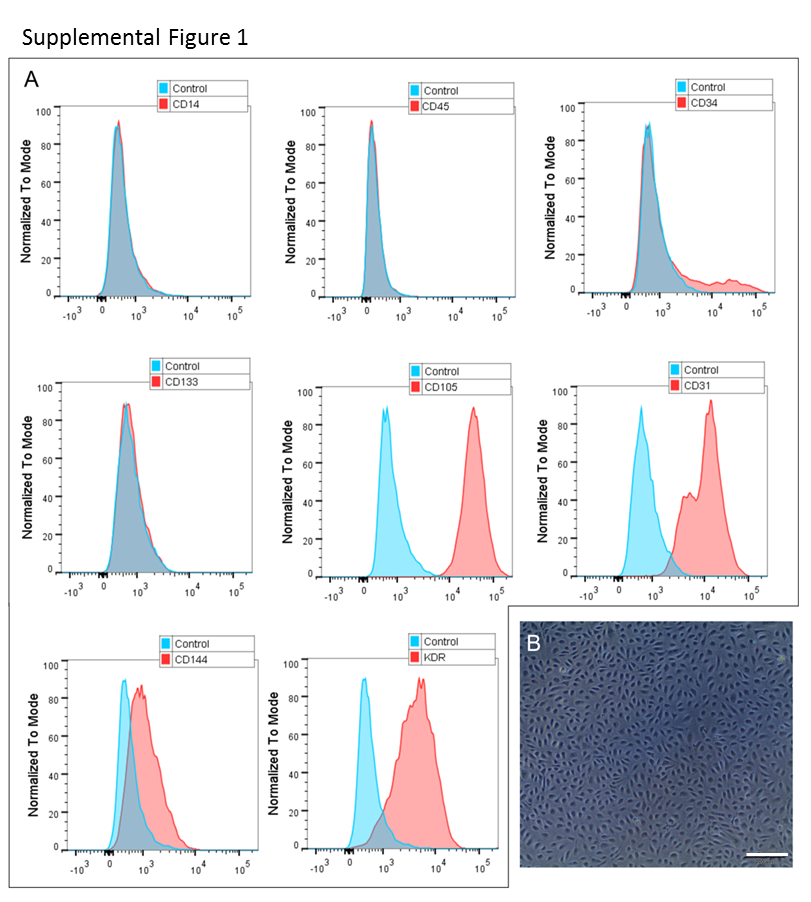

Supplement: Figure S1 — Characterization of ECFCs by (A) flow cytometry of cell type markers and microscopy (B). ECFCs do not express the leukocyte markers CD14 or CD45, retain some CD34 expression but not CD133, and express the endothelial markers CD105 (Endoglin), CD31 (PECAM), CD144 (VE-Cadherin) and KDR (VEGFR-2). A monolayer of ECFCs shows a typical cobblestone pattern in vitro (magnification 4x). (TIF) [file pone.0073161.s001.tif]

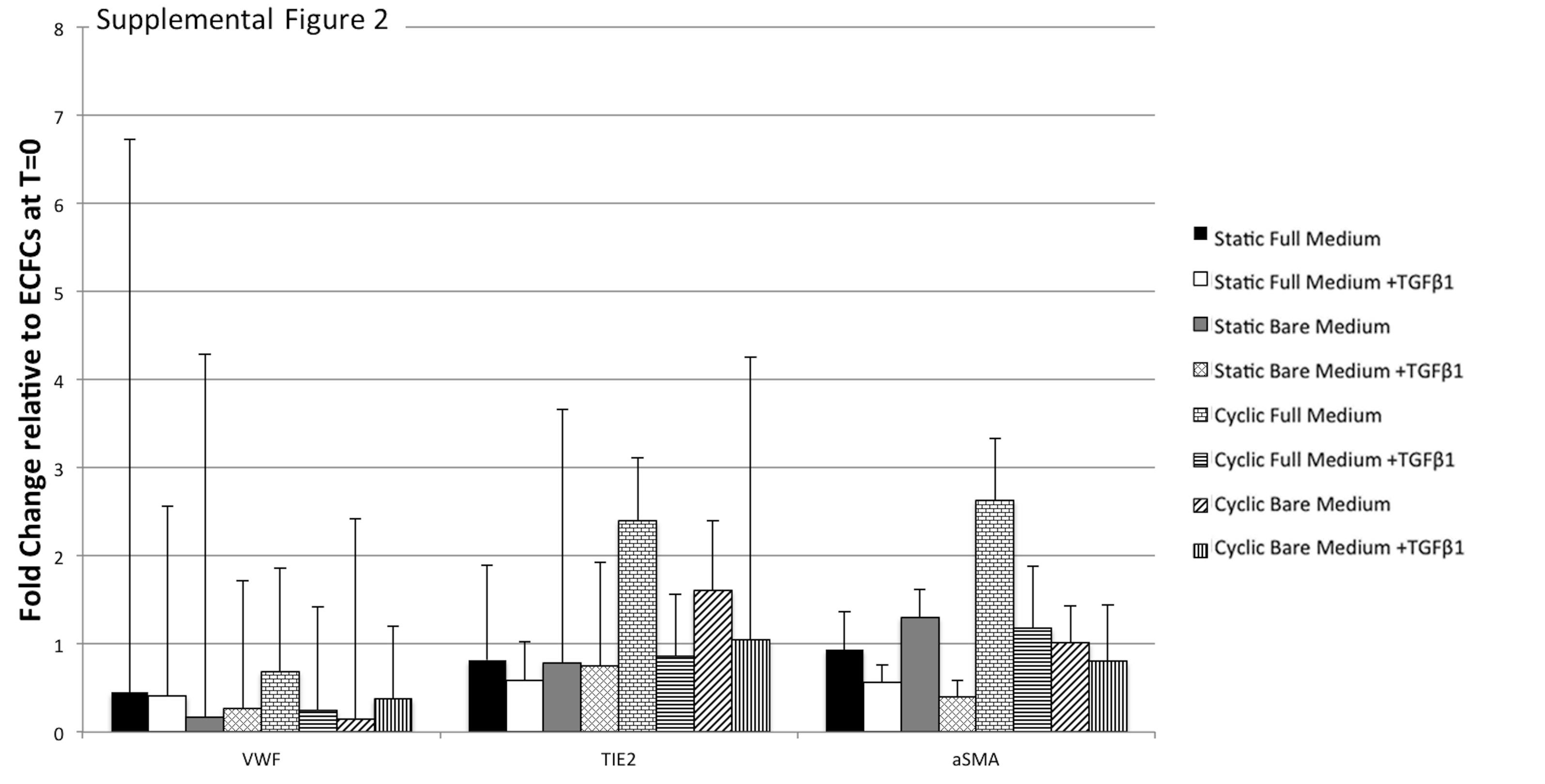

Supplement: Figure S2 — qPCR of ECFCs cultured in 3D in all medium groups under static or cyclic strain. Expression of EndoMT genes is shown relative to ECFCs before seeding into the 3D gels. Medium groups and static or cyclic strain did not result in any significant altering of the gene expression of VWF, TIE2 and αSMA. N = 3 (TIF) [file pone.0073161.s002.tif]

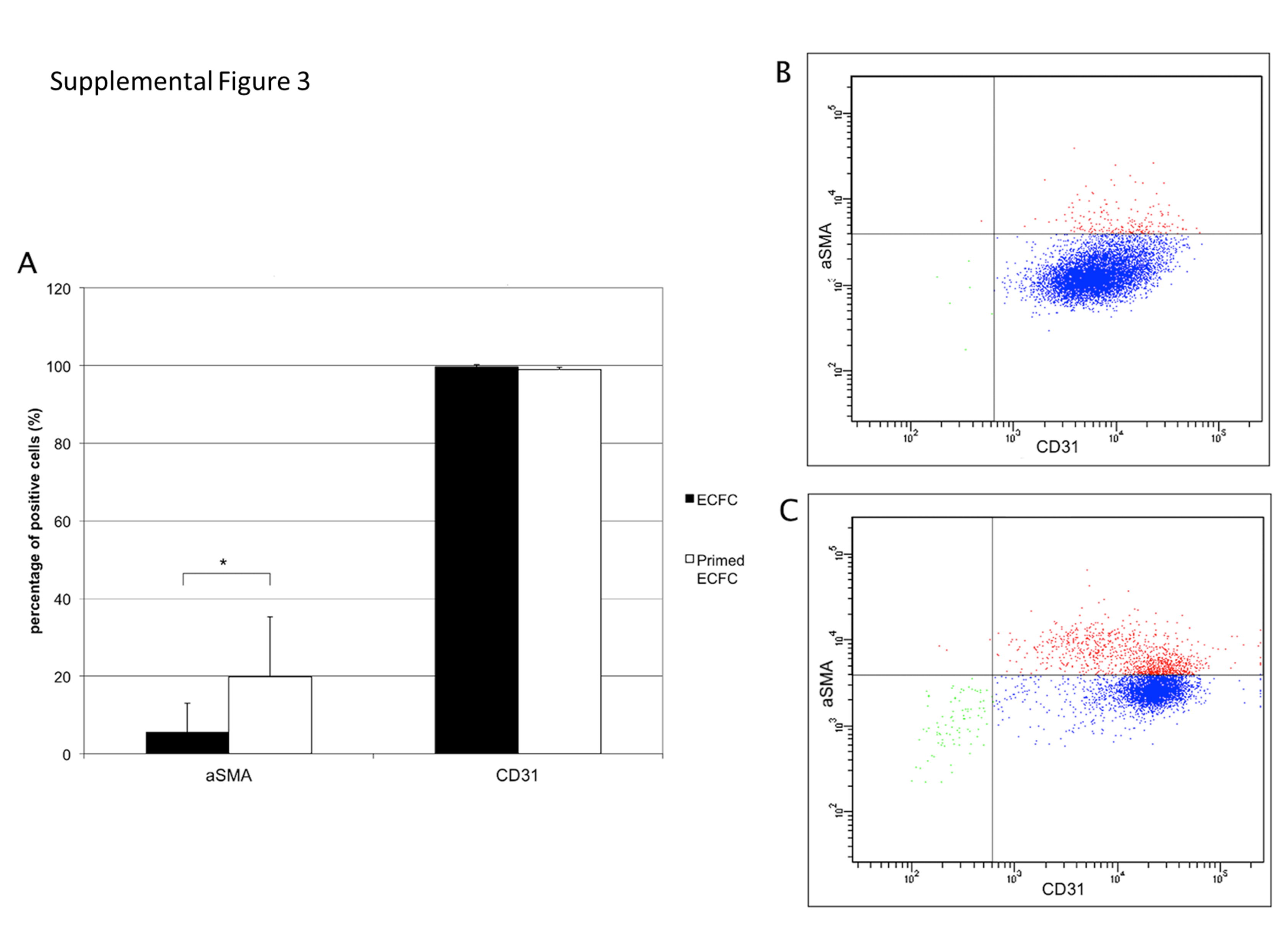

Supplement: Figure S3 — Flow cytometry of ECFCs before and after priming with TGFβ1 and depletion of eGFS for 15 days. (A) The percentage of cells positive for αSMA increased to 20%. The percentage of cells positive for CD31 remained 100%. (B) Representative scatterplots of ECFCs before priming with TGFβ1 and (C) after priming with TGFβ1. (TIF) [file pone.0073161.s003.tif]

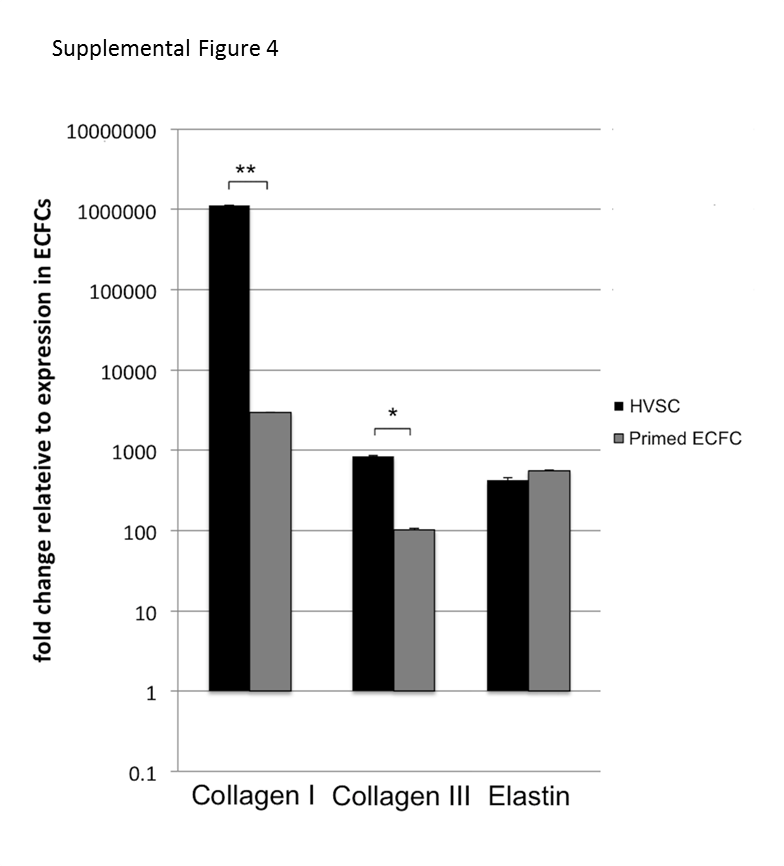

Supplement: Figure S4 — qPCR of HVSCs and pre-treated ECFCs cultured in 2D. Expression of ECM genes is shown relative to ECFCs, cultured in 2D with full medium. Pre-treated ECFCs show an up-regulation of collagen I compared to ECFCs. HVSCs on the other hand, express collagen type I significantly more than pre-treated ECFCs do. No significant differences were found comparing HVSCs with pre-treated ECFCs for collagen III, IV and elastin. ** = p<0.01, N = 3. (TIF) [file pone.0073161.s004.tif]
